# Maggot debridement therapy as primary tool to treat chronic wound of animals

**DOI:** 10.14202/vetworld.2016.403-409

**Published:** 2016-04-25

**Authors:** Vijayata Choudhary, Mukesh Choudhary, Sunanda Pandey, Vandip D. Chauhan, J. J. Hasnani

**Affiliations:** 1 Department of Veterinary Parasitology, Veterinary College, Anand Agricultural University, Anand, Gujarat, India; 2 Department of Veterinary Pathology, Veterinary College, Anand Agricultural University, Anand, Gujarat, India

**Keywords:** chronic wounds, debridement, *Lucilia sericata*, maggots, maggot debridement therapy

## Abstract

Maggot debridement therapy (MDT) is a safe, effective, and controlled method ofhealing of chronic wounds by debridement and disinfection. In this therapy live, sterile maggots of green bottle fly, Lucilia (Phaenicia) sericata are used, as they prefernecrotic tissues over healthy for feeding. Since centuries, MDT is used in humanbeings to treat chronic wounds. Lately, MDT came out as a potent medical aid in animals. In animals, although, this therapy is still limited and clinical studies are few. However, with the increasing antibiotic resistance and chronic wound infections in veterinary medicine, maggot therapy may even become the first line of treatment for some infections. This paper will present a brief discussion of MDT and its role in veterinary medicine that may add one more treatment method to utilize in non-healing wounds of animals and overcome the use of amputation and euthanasia. The objective of this review paper is to assemble relevant literature on maggot therapy to form a theoretical foundation from which further steps toward clinical use of maggot therapy in animals for chronic wounds can be taken.

## Introduction

Maggot debridement therapy (MDT) is a form of therapeutic wound treatment in which sterile or disinfected larvae of certain blowfly species are used to remove non-vitalized tissue, pus, slough, and metabolic wastes on the wound and promote healing [1]. MDT is also known as maggot therapy, biodebridement, larval therapy [2], biosurgery [3,4], biotherapy, and biosurgical debridement [5,6]. The larvae of the sheep blowfly, *Lucilia* (*Phaenicia*) *sericata* are the most widely used species for MDT due to its preference for feeding on necrotic tissues over healthy [6-8]. In this therapy live, sterile fly larvae commonly known as medicinal maggots are applied to the wound to effect debridement, disinfection, and ultimately wound healing [3]. The success rate of MDT was reported in literature ranges from 70% to 80% [6,9,10]. However, use of MDT in veterinary medicine has been neglected but has recently emerged as an effective treatment for chronic wounds of animals. MDT is most commonly used in horses and small animals to treat a different kind of wounds. Maggots are used less often in pets, but some small animal veterinarians are now using MDT for problematic dog and cat wounds [11,12].

### History of MDT

The first observationof maggots and their beneficial effects in wounds were made in 1557 by Ambrose Paré [13,14]. In 1829, Baron D.J Larrey, a military surgeon in Napoleon’s army used the fly larvae in wounds to reduce the threat of bacterial infection and stated that “maggots promoted healing without leaving any damage” [14,15]. Zacharias and Jones were the first, who clinically applied maggots to the wounds during the American Civil War [16]. In 1928, William Baer, an orthopedic surgeon, is credited with the first use of blowfly maggots to treat osteomyelitis wounds in hospital [17]. Maggots were used extensively in hospitals during the 1930s and 1940s, especially in the United States. After the invention of penicillin and better surgical techniques, the interest in MDT gradually disappeared in the early 1940s until 1980s when methicillin-resistant *Staphylococcus aureus* (MRSA) became a problem[10].

With the appearance of antibiotic resistance and increasing problems in treating chronic wounds worldwide, MDT re-emerged as a useful therapy for surgical wounds infected with antibiotic-resistant *S. aureus* [4,18,19]. Afterward in 1989, Ronald Sherman used larvae or maggots to treat decubital ulcers [10].

### Current status of maggot therapy

In 2004, Food and Drug Administration (FDA) granted permission for “the production and distribution of medical maggots” to be marketed as a medical device for wound care [11,19-21]. As MDT has approved by FDA, maggots are produced aseptically and delivered by commercial companies to wound care centers and hospitals worldwide and regulates medicinal maggots as a medical device [19]. Currently, there are 12 laboratories in 20 countries dispensing maggots at low cost [22], but these larvae are sometimes sold at a very high price to veterinarians who occlude their use for economic reasons [23].

## Biology of Blow Fly

The common green bottle fly, *L. sericata* (Diptera: Calliphoridae), formerly *Phaenicia sericata*, belonging to the Diptera orderof insects, is a necrophagous fly which feeds on carrion, feces and garbage, and plays an important role in forensic, veterinary, and medical science [24,25]. *L. sericata* is commonly known as blow fly, sheep blow fly and green bottle fly, which is commonly found in cool-temperate habitats [26]. Adult flies of *L. sericata* are metallic green or copper green [27]. The average longevity of an adult female is about 7 days [1,28]. In forensic science, the larvae or maggots are used as a biological indicator in the determination of post-mortem interval [29]. In the field of veterinary medicine, *L. sericata* is characterized as a facultative ectoparasite responsible for primary cutaneous myiasis. Medical treatment using maggots can help to heal chronic injuries that do not respond to conventional treatments [25] and in more acute wounds, especially when surgical debridement is difficult to perform because of the location of the wound and the anatomical structures involved.

### Lifecycle

Life cycle begins when adult female flies deposit clusters of eggs (about 2000-3000) on animal corpses, infected wounds of humans or animals and excrement, where they hatch in 18-24 h [1,28,30]. Eggs are usually white, elongated with one end tapered slightly, and hatch in 4-7 days [27]. Development of larvae includes three stage, i.e., 1^st^, 2^nd^ and 3^rd^ instars. The size of 1^st^ and 3^rd^ instar larvae (mature larvae) are 1-2 and 10 mm, respectively [29,30]. Mature larvae are smooth, yellowish-white, conical shaped, and have both anterior and posterior spiracles. After maturation of 3^rd^ instar larvae, they leave the host or carrion and burrow into the soil or substrate surrounding it where they pupate after 7-10 days. The pupal case is hard, reddish-brown to black. After a pupal period of 6-14 days the adult fly emerges from the puparium, it reproduces and starts the cycle all over again [31]. The life cycle of *L. sericata* depends on ambient temperature, but normally completes within 4-6 weeks [32].

### Production of disinfected larvae of L. (Phaenicia) sericata

The larvae of *L. sericata* are relatively small (1-2 mm) when they are applied for woundmanagement. After feeding on necrotic tissue in the moist environmentof wounds, they can grow up to 1 cm in 2-3 days [10], after that they will be changed for new ones [33]. For the production of sterile maggots in the laboratory, eggs are collected from gravid females of *L. sericata* (laboratory colony). These eggs are disinfected with a dilute phenol disinfectant (3% Lysol Brand Disinfectant, Reckitt and Coleman Inc.) or 0.525% sodium hypochlorite [12], transferred to sterile vials and left at room temperature overnight. About 500-1000 larvae hatch in each vial and are available for clinical use after quality assurance [12,34].

Maggots are microbiologically tested before use, to certify that no infection will be induced by MDT [35]. The maggots are shipped in a temperature controlled package and are approximately 1-day-old when they arrive for the treatment [36]. They should be used as soon as possible, but essential maturation can be detained by storing at refrigerator temperature (4-8°C) for 2-5 days [34-37]. In case maggots transport is expected to take 2 days then it is worth ordering more as only half of the maggots are alive and able to debride on delivery after a 2 days transport [23]. Maggots are supplied either in free range or in biobag (contained) by constructed laboratories owned by companies such as “BioMonde (formerly ‘ZooBiotic’) Ltd.” based in Brigend, South Wales [5,38].

## Mechanism of Action

Medicinal maggots (larvae) have the following beneficial effects on a wound: Tissue debridement, disinfection, stimulation of healing, biofilm inhibition, and eradication [5,28]. *L. sericata* larvae do not penetrate tissues deeply as they depend on aerobic conditions. Thus the larvae interrupt wound surfaces by crawling around using their hook-like mouthparts [39].

Maggots move over the surface of the wound secreting a powerful mixture of digestive enzymes such as carboxypeptidases A and B, leucine aminopeptidase, collagenase, and serine proteases (trypsin-like and chymotrypsin-like enzymes metalloproteinase, and aspartyl proteinase) [8]. These proteolytic enzymes, break down the dead tissue, liquidizing it and ingest the resulting liquefied material [19]. Chymotrypsin-like serine proteinase plays a significant role in the digestion of wound matrix and effective debridement through degradation of extracellular matrix components laminin, fibronectin, and collagen Types I and III [1]. Mechanical action of maggots and secretion of proteolytic enzymes helps in efficient tissue debridement. There is no danger to healthy tissue as these enzymes are neutralized when they come into contact with intact tissue. In this way, they remove cellular debris, dead contaminated tissue, microbes, and foreign material [39,40].

Secretions of maggots increase the wound pH through secretion of sodium bicarbonate, thereby inhibiting the growth of bacteria [37,39]. Maggots also ingest and digest bacteria within the devitalized tissue in the wound, which are killed in their gut [41]. It was shown that excretions/secretions contain bactericidal components with healing properties, such as allantoin, urea, phenylacetic acid, phenylacetaldehyde, and calcium carbonate, especially against MRSA (the most common organism in wounds) [8,28].

*In vitro* studies have shown thatmaggot secretions can disrupt biofilms created by *Staphylococcus epidermidis*, *S. aureus* and *Pseudomonas aeruginosa*, and maggots also decrease surface bacterial load by ingesting *Escherichia coli* [1,42]. Maggot secretions have chemotactic factors and fatty acids that affect migration of fibroblasts and induce granulation tissue formation subsequently along with tissue oxygenation [37,40].

Therefore, MDT helps to separate necrotic tissue from the underlying bed, kills microorganisms, disrupts biofilm, and hastens wound healing through a broad range of factors including leukocyte adhesion, growth factor production, collagen production, increased angiogenesis, increased macrophage responsiveness, increased fibrinolysis, and increased nitric oxide levels [43].

## Techniques of Maggot Application

Maggot therapy is a controlled method in which safe and effective species and strains of maggots are selected and made germ-free by chemical disinfection. These maggots are called as medicinal maggots and apply on the wound with special dressings that prevent them from leaving the wound unescorted [5]. Maggots of *L. sericata* are used in this technique as they are non-invasive [44]. The maggots available in the United States belong to the (currently named) LB-01 strain of *Phaenicia* (*Lucilia*) *sericata* [5].

Before application, the wound is cleaned (without the use of antiseptics), with normal salt solution or sterilized water to remove the grease and dirt [45]. Maggots are applied on the wound in two-ways, i.e., free range (direct contact method) and biobag (indirect contact method) dressing [23,46,47]. In free range dressing, maggots are applied directly to the wound for 3 days, and allowed to roam freely over [47,48], the surface seeking out areas of slough or necrotic tissue due to which maggots can escape out from the wound site. In biobag dressing, the maggots are enclosed in net pouches containing pieces of hydrophilic polyurethane foam, which are placed directly upon the wound surface for 5 days, so that maggots cannot escape dressing, and the structure of the mesh enables the proteolytic secretions to reach the necrotic tissue, and maggots can still aid in debridement [46]. It also prevents the sensation of tingling due to movement of maggots on the normal skin [45]. Maggots are obligate air breathers, so the dressing must also allow fresh air to enter the area, and let the liquefied necrotic tissue to drain freely from the wound [8,36,49].

Biobag method of maggot application is less complicated as it requires less experience, saves labor, time and resources saving during dressing changes, and is less painful than free range dressing [37]. The disadvantage of the biobag method is that debridement is less effective as the maggots cannot use mouth hooks through the biobag [46]. According to Steenvoorde *et al.*, biobag have a significant negative impact on the successful outcome of MDT [9]. In another study, Blake *et al.*, compared the two techniques and found that debridement efficiency appears to be similar [37]. Statistical analysis revealed no difference between the use of freely crawling maggots and maggots in a biobag regarding the total amount of debrided tissue after 3 or 4 days of treatment.

Maggots are applied to the wound at a dose of 5-10 maggots/cm^2^ of wound surface area in human medicine [5,50,51]. In veterinary medicine, use of 5-10 maggots/cm² of wound surface area [12,23] and 8-12 maggots/cm² has been reported [52]. The number of maggots applied to the wound depends on the amount of necrotic tissue, wound depth, and width of wound area [23,30]. According to Blake *et al*. a standard dose of 100 maggots can debride 50 g of necrotic tissue during one treatment cycle while taking larval death in the wound into account [37]. In a study on horses, the surface area was multiplied by the depth of the wound for the deeper wound (>2 cm) [23].

During the first 2 days, there is a slight amount of odor due to the phagocytic activity of maggots, later on this odor ceases, wound become alkaline and granulation tissue starts developing across the wound. Usually, wounds have an acidic reaction which turns into alkaline, 24 h after application of maggots. Alkaline reaction is helpful in the sterilization of the wound and killing of the bacteria [45]. Maggots are left within their dressing (cage dressing) for 3-4 days, after that, maggots are satiated and can no longer remove any necrotic tissue [44,53]. At this point, maggots should be removed, the wound should be thoroughly washed out with physiological saline solution, and a new batch of maggots should be applied to the wound until the wound is completely debrided [44,45]. At the end of the second application, the wound is completely filled with granulation tissue [45]. This therapy is very effective for pressure ulcers (bed sores) [44], venous stasis ulcers, diabetic foot ulcers [44], non-healing traumatic and post-surgical wounds, burns, and gangrene in human medicine [8,54].

## MDT in Animals

For centuries, MDT has been used to treat chronic infections in humans [22,23,55], but rarely has it been reported in the treatment of infections in animals [56]. Recently, MDT regressed as a powerful and effective therapy for incurable wounds of animals. This therapy is most effective for deep penetrating wounds that have hard to reach soft tissue necrosis such as infections of the palmer regions of the foot, navicular apparatus, digital cushion, and coffin joint [35]. At present, many veterinarians use MDT to treat problematic wounds due to the non-traumatic debridement and disinfection of blowfly maggots in humans [5,57].

Maggot therapy has been reported to be used successfully in a wounded bull [58], two donkeys [56,59], two ponies [23], mule and horses [53]. The first study of MDT in donkeys was carried out by Bell in 2001 [56] and in horses was done by Jurga and Morrison 2004 [60]. In horses, MDT is used in the management of supraspinous bursitis [23], septic navicular bursitis, complicated laminitis [35,61] infected abscess with pedal bone osteomyelitis and other hoof diseases [60]. Kociova *et al*. reported the use of MDT in 6 sheep with 3 cases of acute inflammation of the interdigital skin and 4 cases of purulent inflammation of the pododerm. Four sheep exhibited a marked improvement and initiation of healing already after the first application, two sheep required additional treatment during which 100-120 new larvae were applied beneath the keratin hoof capsule. The effect of a single application for 3-6 days was evaluated. Debridement was rapid and selective. The treatment was well tolerated by animals. New layers of healthy tissue were formed over the wounds [52]. Morrison *et al*. treated 41 cases of coffin bone osteomyelitis, 18 cases of chronic laminitis, 8 cases of septic navicular bursa, 4 cases of chronic distal interphalangeal joint sepsis, 3 cases of canker, 2 cases of non-healing foot ulcers (palmer region of the foot), 1 case of acute caudal coffin bone rotation and 1 case of necrosis of collateral cartilage (Quittor) in horse of both sexes with MDT [35]. Once stability is achieved, maggot therapy has been able to rid the infection. None of the cases described were euthanized for failure to treat the infection. The only side effect of maggot therapy is the occasional patient may experience irritation or itching at the wound site. The movement of the larvae within the wound may be a cause for this [40]. A total of 20 cases were treated with MDT in which 7 cases of small animals (2 dogs, 4 cats, 1 rabbit) and 13 cases of horses [12]. In this study, maggot therapy was associated with limb salvage in three of the five canines and felines that were expected to require amputation or euthanasia. One rabbit was treated with maggot therapy over the entire hock, due to a septic wound. One cycle of maggot debridement was applied, with significant debridement. No adverse events were attributed to maggot therapy for any of these cases. It should be noted, however, that two animals with serious infections succumbed to sepsis during therapy, and the septic rabbit died 4 days following maggot therapy.

Later on, Sherman *et al.*, used MDT to treat a 6-year-old horse with an extensive laceration of the left proximal hind limb, a newborn foal suffering from an obliterative vasculitis involving the hoof, a 9-year-old mare with puncture wound involving the navicular bursa, digital flexor tendon, coffin joint and digital cushion of the right hind limb, a 14-year-old Arabian stallion with a puncture wound to the lateral heel. Following maggot therapy, all infections were eradicated or controlled, and only one horse had to be euthanized. No adverse events were attributed to maggot therapy for any of these cases, other than presumed discomfort during therapy [53]. Lepage *et al*. treated 41 equids (35 horses, 4 donkeys and 2 ponies of all ages and both sexes) by MDT with various lesions *viz*., septic navicular bursa, fistulous withers in a donkey, keratoma, septic pedal bone osteitis, chronic proliferative wound dorsal left hind cannon bone, chronic proliferative wound proximo plantar aspect right hind tarsus, soft tissue abscess (1 buttock, 3 neck, 1 scrotum, 1 prepuce), various acute or subacute limb laceration, chronic tuber coxaefracture with fistulization and MRSA infected wounds [23]. In 38 cases a favorable outcome was reached in <1 week. In all cases, debridement, disinfection and enhanced healing were observed. In 3 cases, complete healing of the wound failed to occur. In one of these cases, healing occurred uneventfully after a bony sequestrum was removed. In 2 other horses, squamous cell carcinoma and melanoma were involved in chronic infected wounds and complete healing was not achieved because of recurrence of underlying tumors [23].

MDT were found successful in small animals and horse with complete healing in wounds that had already failed to respond to conventional medical and surgical therapy [12,34,53]. This therapy assists with wound healing by debridement of necrotic wounds [23,34,52,53], disinfection [23,53] and granulation tissue formation [23,52] occur after 3 days of application [12]. If complete debridement of the wound does not occur, the second application of maggots is required [12]. In some cases of small animals and horses, light surgical debridement was performed along with MDT to remove debris and dry necrotic tissue [23,44,53]. MDT has potent antibacterial effects as it controls difficult infections such as MRSA or other multi-antibiotic resistant bacteria [23,56]. It is an effective way to prevent the establishment of serious infections including complicated and deep lacerations, abscesses, abdominal wound dehiscence, and infections even in the presence of internal fixation [23], severe distal phalanx displacement and solar penetration [8]. It is reported that maggot therapy was associated with limb salvage in three of the five canines and felines that were expected to require amputation [12]. Other treatments, such as systemic antibiotic injections, local perfusion with antibiotics and use of disinfectants (e.g., dilute betadine or chlorine dioxide) before application of maggot therapy, have no effect on the viability of the maggots [8]. In some cases, antibiotic treatment was not used with MDT for the better assessment of the efficacy of this therapy [12]. Occasionally, general anesthesia was needed to control the animal and for the application of the dressing in a proper way [12]. Few animals died or were euthanized due to the severity of pre-existing diseases, and not due to treatment failure [12,33].

Maggot therapy is a safe and useful technique for debriding and disinfecting some severe wounds and has emerged as an alternative to amputation in dogs and cats. Progressively, small animal veterinarians are turning to MDT to treat problematic wounds [12].

## Contraindications and Side Effect of MDT

Adverse reactions to MDT usually does not happen [61,62] and only side effects observed are irritation, itching and hypersensitivity [34] at the wound site. Sometimes it also causes pain, discomfort and malodor at the first change of dressing [38,44,62,63]. In the case of horses, discomfort was observed by the movement and stamping of the limb and repeated rubbing of the bandage [23,34]. No adverse effects have been identified in small animals [12].

Maggots should be avoided to treat dry wounds as they require a moist environment [22,36]. Maggot therapy should not be used in open wounds of body cavities or sinuses, fistulae, wounds in close proximity to large blood vessels [36,46], wounds close to the brain, wounds with neoplasia [23] and wounds with large necrotic areas such as a large area of osteomyelitis or a sequestrum [23,44]. Patients with rapidly advancing infection [46] and sepsis should not initially be treated with MDT [9].

During application of maggots, excessive pressure on the wound can kill maggots in that area, leading to uneven wound debridement. Maggots should be changed every 4^th^ day [34] as they have limited “shelf-life” and need to be applied soon after delivery [64,65]. The wound should be entirely filled with the maggots so that every part is attacked by the maggots at the same time [45]. Large number of maggots for a short period should be used rather than a small number for an extended period [46].

In veterinary medicine, use of MDT was limited by the relatively high cost of treatment, time required for shipping (usually 24-48 h after ordering) and the fact that a portion of maggots failed to survive on arrival [12,23]. Another common problem was the time and effort involved in applying the dressings in such a way that they are not removed by the animal [12].

## Conclusion and Future Aspects

MDT is frequently used in human medicine although this therapy is still limited in animals and clinical studies are few. However, with the increasing antibiotic resistance and chronic wound infections in veterinary medicine, maggot therapy may even become the first line of treatment for some infections. Clinical studies evaluated that MDT is more efficient and safe technique for chronic wounds as compared to conventional therapy. Maggots are supplied by licensed laboratories in sterile bottles, which make it very safe, efficient, and easy method of healing for chronic wounds and prevents secondary bacterial infections. There is an overwhelming need for improved wound care in countries which are under-provided for medical veterinary facilities. In veterinary medicine, further clinical studies are needed in several fields, including establishing a number of maggots required for safe and efficient treatment and identification of adverse effect during treatment. There is also a need to assure the safety and efficiency of the treatment in small animals, increase awareness in owners and encourage veterinarians to take the first steps toward using MDT in dogs, cats and horses, and can help to make MDT as accessible. In addition, MDT will add one more treatment method to utilize in non-healing wounds of animals and overcome the use of amputation and euthanasia.

## Authors’ Contributions

This topic was studied and reviewed by VC and MC and revised by SP and VDP under the guidance of JJH. All authors read and approved the final version of the article.
